# Supplementary material for: MITF and TFEB cross-regulation in melanoma cells
Source: PLoS One. 2020 Sep 3;15(9):e0238546. doi: 10.1371/journal.pone.0238546 (PMC7470386; doi:10.1371/journal.pone.0238546)
Supplement: S1 Fig — (A) RNA expression of MITF and TFEB after overexpression of GFP-tagged MITF (+) in 501Mel cells compared to empty vector control. (B) RNA expression of MITF and TFEB after overexpression of GFP-tagged MITF (+) or TFEB in Skmel28 cells compared to empty vector control. (PDF) [file pone.0238546.s001.pdf]

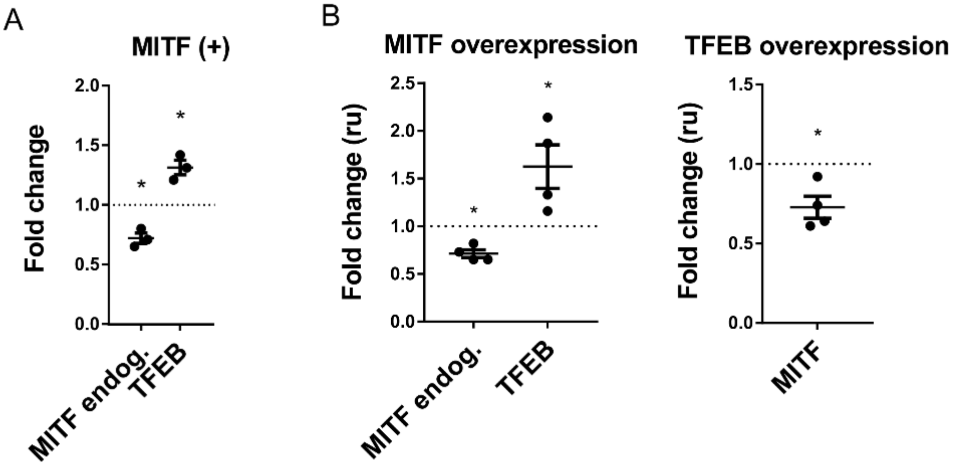

Figure S1. MITF and TFEB modulate each other's expression upon overexpression in 501Mel and Skmel28 cells. (A) RNA expression of *MITF* and *TFEB* after overexpression of GFP-tagged MITF (+) in 501Mel cells compared to empty vector control. (B) RNA expression of *MITF* and *TFEB* after overexpression of GFP-tagged MITF (+) or TFEB in Skmel28 cells compared to empty vector control.
