# Supplementary material for: MITF and TFEB cross-regulation in melanoma cells
Source: PLoS One. 2020 Sep 3;15(9):e0238546. doi: 10.1371/journal.pone.0238546 (PMC7470386; doi:10.1371/journal.pone.0238546)
Supplement: S2 Fig — The expression of MITF and TFEB as determined by RT-qPCR after siRNA knockdown of each factor, compared to control siRNA in Skmel28 cells; two independent experiments are shown. Bars represent SEM. * indicates significance at p<0.05. (PDF) [file pone.0238546.s002.pdf]

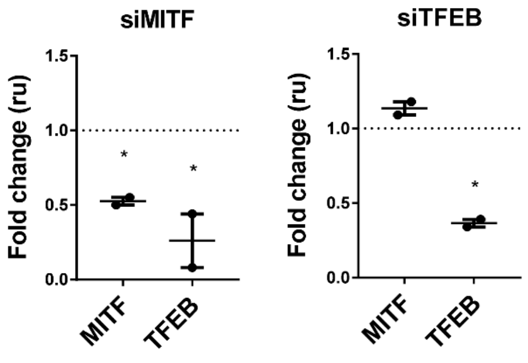

Figure S2. siRNA-mediated knockdown of MITF modulates endogenous *TFEB* expression in Skmel28 cells. The expression of *MITF* and *TFEB* as determined by RT-qPCR after siRNA knockdown of each factor, compared to control siRNA in Skmel28 cells; two independent experiments are shown. Bars represent SEM. * indicates significance at $p<0.05$.
