# Supplementary material for: MITF and TFEB cross-regulation in melanoma cells
Source: PLoS One. 2020 Sep 3;15(9):e0238546. doi: 10.1371/journal.pone.0238546 (PMC7470386; doi:10.1371/journal.pone.0238546)
Supplement: S3 Fig — HEK293T cells were transiently co-transfected with a p3XFLAG-CMV-14 construct with or without R214-217A MITF-M (empty vector, EV) and luciferase constructs and assayed for luciferase activity after 24h. Luminescence signal is expressed as fold change over an empty reporter for Tyrosinase and a reporter containing the element from TFEB intron 1 in front of a minimal SV40 promoter followed by luciferase. Error bars represent the SEM of three experiments. * indicates significance at p<0.05. (PDF) [file pone.0238546.s003.pdf]

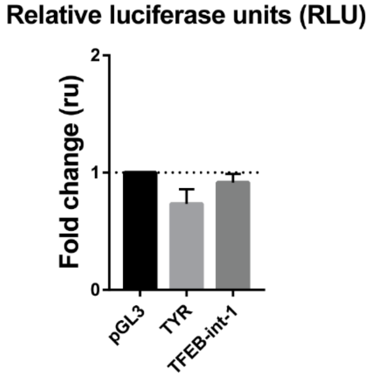

Figure S3. R214-217A MITF fails to transactivate *TYR* or *TFEB* regulatory elements. HEK293T cells were transiently co-transfected with a p3XFLAG-CMV-14 construct with or without R214-217A MITF-M (empty vector, EV) and luciferase constructs and assayed for luciferase activity after 24h. Luminescence signal is expressed as fold change over an empty reporter for *Tyrosinase* and a reporter containing the element from *TFEB* intron 1 in front of a minimal SV40 promoter followed by luciferase. Error bars represent the SEM of three experiments. * indicates significance at $p<0.05$.
