# Supplementary material for: MITF and TFEB cross-regulation in melanoma cells
Source: PLoS One. 2020 Sep 3;15(9):e0238546. doi: 10.1371/journal.pone.0238546 (PMC7470386; doi:10.1371/journal.pone.0238546)
Supplement: S4 Fig — Immunofluorescence images of human Skmel28 cells after treatment with vehicle (DMSO) or an mTOR inhibitor (Torin-1, 1 μM, 3 hours), showing endogenous TFEB and MITF proteins in green. (PDF) [file pone.0238546.s004.pdf]

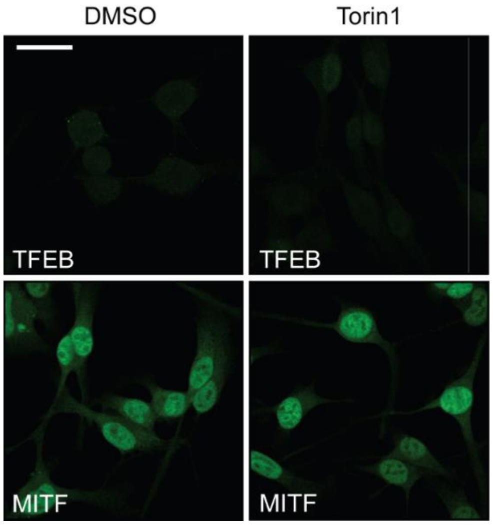

Figure S4. mTOR signaling affects the subcellular localization of MITF in Skmel28 cells. Immunofluorescence images of human Skmel28 cells after treatment with vehicle (DMSO) or an mTOR inhibitor (Torin-1, 1 μM, 3 hours), showing endogenous TFEB and MITF proteins in green.
