# Supplementary material for: MITF and TFEB cross-regulation in melanoma cells
Source: PLoS One. 2020 Sep 3;15(9):e0238546. doi: 10.1371/journal.pone.0238546 (PMC7470386; doi:10.1371/journal.pone.0238546)
Supplement: S1 Table — (PDF) [file pone.0238546.s005.pdf]

Table S1. MITF, TFEB and TFE3 but not TFEC are expressed in 501Mel and Skmel28 cells

	MITF	TFEB	TFE3	TFEC	β-actin
**501Mel**	126,938	4,956	1,098	71	316,702
**Skmel28**	101,400	963	1,286	70	322,223

Gene expression values generated using a microarray platform for MITF, TFEB, TFE3, TFEC and Actin in 501Mel and Skmel28 melanoma cells.
