# Supplementary material for: MITF and TFEB cross-regulation in melanoma cells
Source: PLoS One. 2020 Sep 3;15(9):e0238546. doi: 10.1371/journal.pone.0238546 (PMC7470386; doi:10.1371/journal.pone.0238546)
Supplement: S2 Table — (PDF) [file pone.0238546.s006.pdf]

Table S2. Gene-specific primers used for RT-qPCR

Gene target		Primer sequence
**β-Actin**	FW	5’-AGGCACCAGGGCGTGAT-3‘
	REV	5’-GCCCACATAGGAATCCTTCTGAC-3‘
**RPLP0**	FW	5’-CACCATTGAAATCCTGAGTGATGT-3‘
	REV	5’-TGACCAGCCCAAAGGAGAAG -3‘
**MITF (+/-)**	FW	5’-CGACAGAAGAAACTGGAGCAC-3‘
	REV	5’-AAATCTGGAGAGCAGAGACCC-3‘
**MITF (+)**	FW	5’-ATGGAAACCAAGGTCTGCCC-3‘
	REV	5’-GGGAAAAATACACGCTGTGAGC-3‘
**MITF 3’UTR**	FW	5’-GGGATCCAAACTGGAAGACA-3‘
	REV	5’-AGGAAGCAGTTTGTGCGAAT-3‘
**TFEB**	FW	5’-AAGGAGCGGCAGAAGAAAGA-3‘
	REV	5’-CCAACTCCTTGATGCGGTCA-3‘
**TFE3**	FW	5’-CAGCTGCTCAGCCTGAACTC-3‘
	REV	5’-CTTGAGCGAAGGGGTAAGGG-3‘
