# Supplementary material for: MITF and TFEB cross-regulation in melanoma cells
Source: PLoS One. 2020 Sep 3;15(9):e0238546. doi: 10.1371/journal.pone.0238546 (PMC7470386; doi:10.1371/journal.pone.0238546)
Supplement: S3 Table — (PDF) [file pone.0238546.s007.pdf]

Table S3. Primers used for cloning and mutagenesis of plasmid constructs

Construct name		Primer sequence
**pBac-pEGFP**	FW	5’-TAATTAACGCGTTGAACCGTCAGATCCGCTAG-3‘
	REV	5’-GGCTGATTATGATCTAGAGTCG-3‘
**pBac-MITF-M-FLAG-HA**	FW	5'-TAATTGAATTCCGGTACCAGTCGACTCTAGA-3'
	REV	5'-TAATTACTAGTCTTGTCATCGTCATCCTTGT-3'
**MITF-M-R214-217A**	FW	5'-GCTGCTTTTAACATAAACGACCGC-3'
	REV	5'-AGCAGCTTCAATCAAGTTGTGATTGTC-3'
**TFEB-intron-1**	FW	5’-ACTGTTTGAGGACCCACAG-3‘
	REV	5’-AGTGCTTGGCCTAGTCAG-3‘
**TFEB-intron-1-scrambled**	FW	5’-TTATGTCCTCATGACCTTCCCC-3‘
	REV	5’-AGGGCCTGTCTGGAGAGCCCCT-3‘
