# Supplementary material for: MITF and TFEB cross-regulation in melanoma cells
Source: PLoS One. 2020 Sep 3;15(9):e0238546. doi: 10.1371/journal.pone.0238546 (PMC7470386; doi:10.1371/journal.pone.0238546)
Supplement: S4 Table — (PDF) [file pone.0238546.s008.pdf]

Table S4. miRNA sequences and primers used for the MITF knockdown cell lines

**miR-NTC**	
**Mature miRNA**	**AAATGTACTGCGCGTGGAGAC**
FW	5’-GAAATGTACTGCGCGTGGAGACGTTTTGGCCACTGACTGACGTCTCCACGCAGTACATTTCA-3‘
REV	5’-AAATGTACTGCGTGGAGACGTCAGTCAGTGGCCAAAACGTCTCCACGCGCAGTACATTTCAG-3‘
**miR-MITF-X2**	
**Mature miRNA**	**AAAGGTACTGCTTTACCTGCT**
FW	5’-GAAAGGTACTGCTTTACCTGCTGTTTTGGCCACTGACTGACAGCAGGTAGCAGTACCTTTCA-3‘
REV	5’-AAAGGTACTGCTACCTGCTGTCAGTCAGTGGCCAAAACAGCAGGTAAAGCAGTACCTTTCAG-3‘
**miR-MITF-X8**	
**Mature miRNA**	**TAAGATGGTTCCCTTGTTCCA**
FW	5’-GTAAGATGGTTCCCTTGTTCCAGTTTTGGCCACTGACTGACTGGAACAAGAACCATCTTACA-3‘
REV	5’-TAAGATGGTTCTTGTTCCAGTCAGTCAGTGGCCAAAACTGGAACAAGGGAACCATCTTACAG-3‘
