# Supplementary figures and images for: MITF and TFEB cross-regulation in melanoma cells
Source: PLoS One. 2020 Sep 3;15(9):e0238546. doi: 10.1371/journal.pone.0238546 (PMC7470386; doi:10.1371/journal.pone.0238546)

Figure 2C.  
501 Mel cells

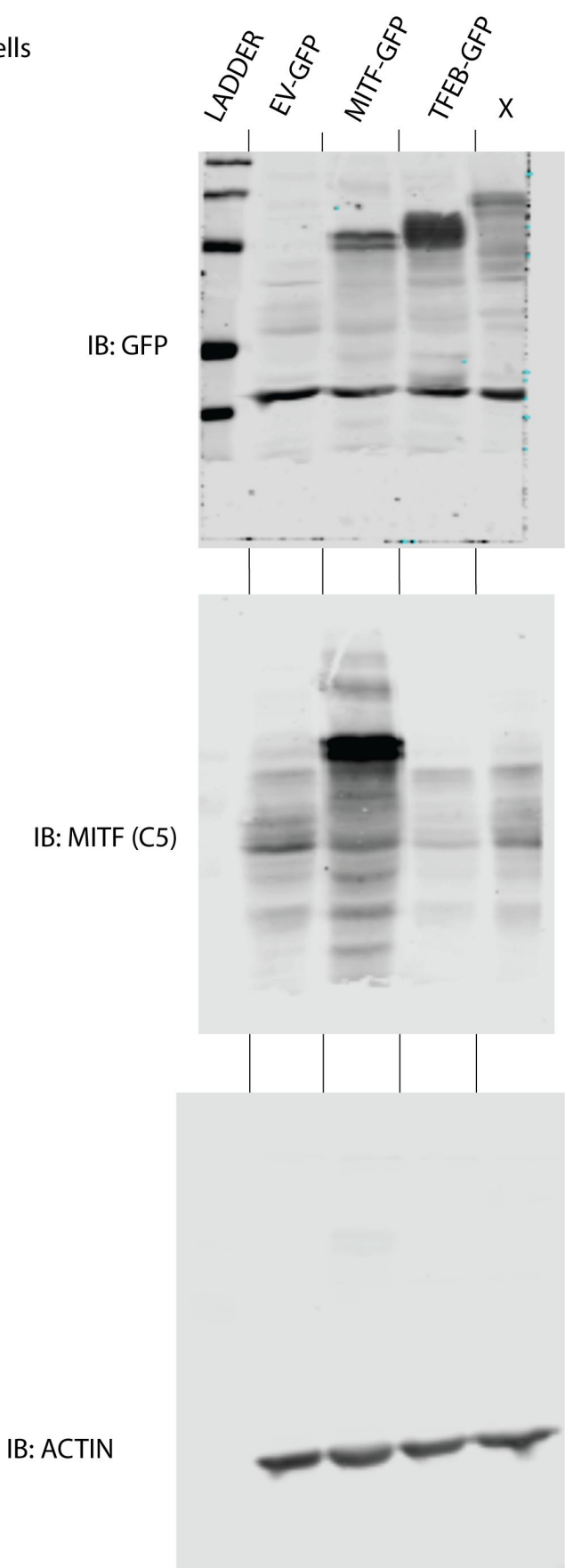

Figure 2D.  
501 Mel cells

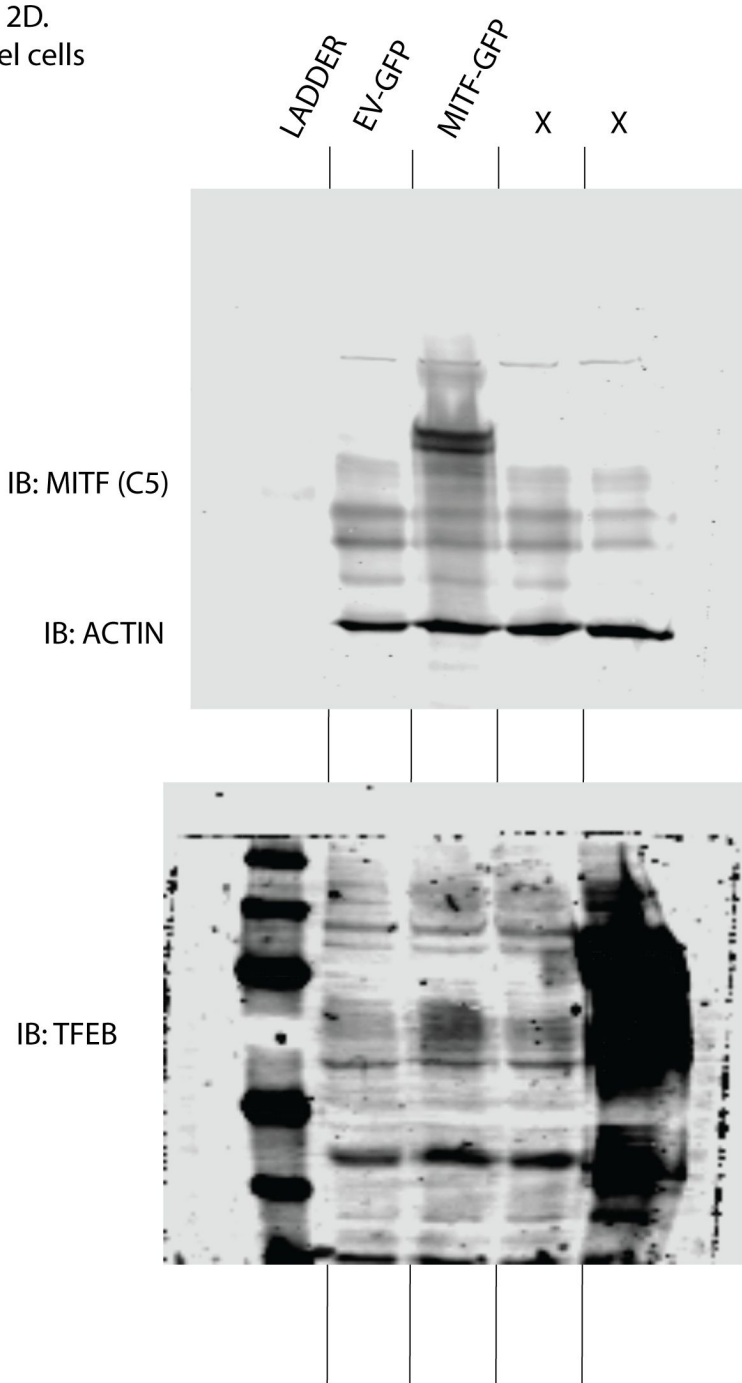

Figure 2C.  
501 Mel cells

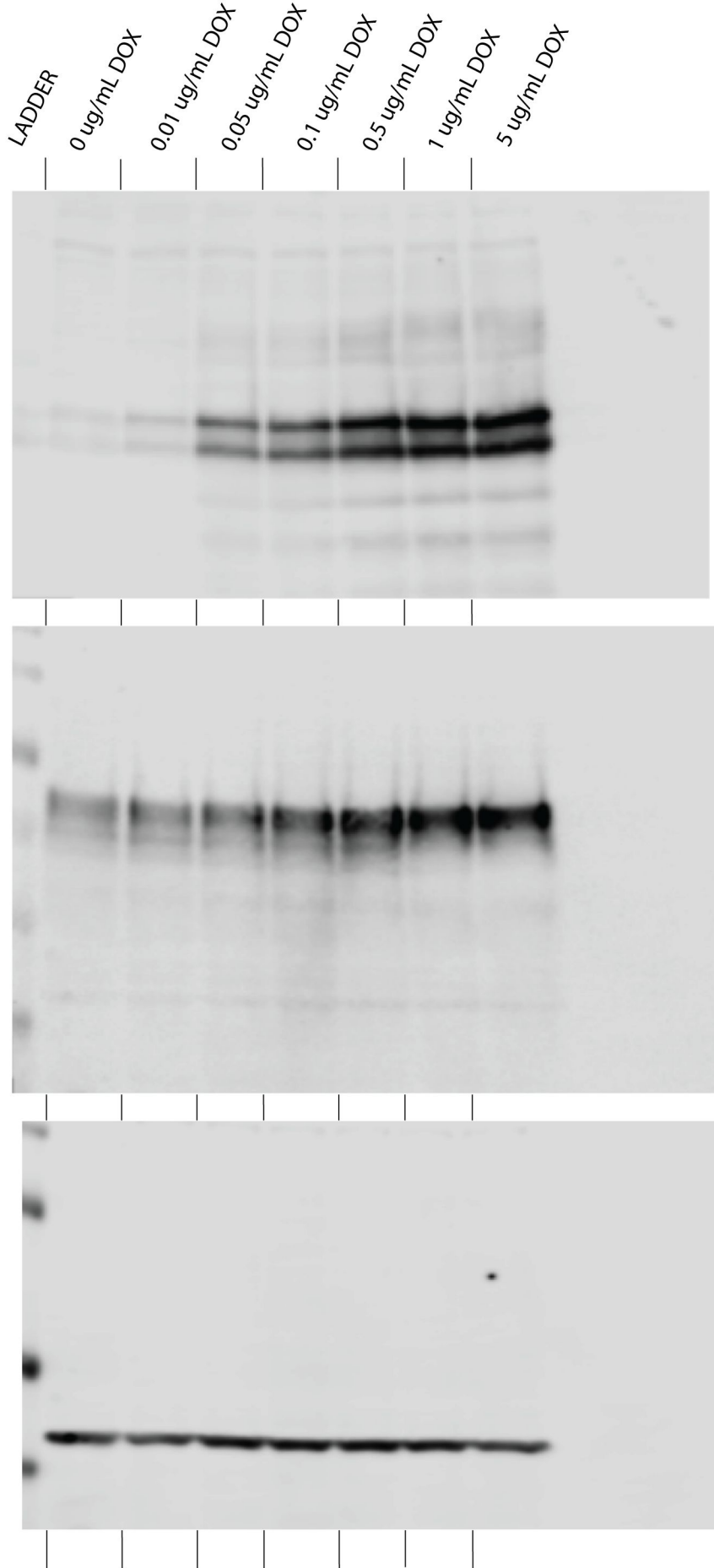

Figure 3B.  
501 Mel cells

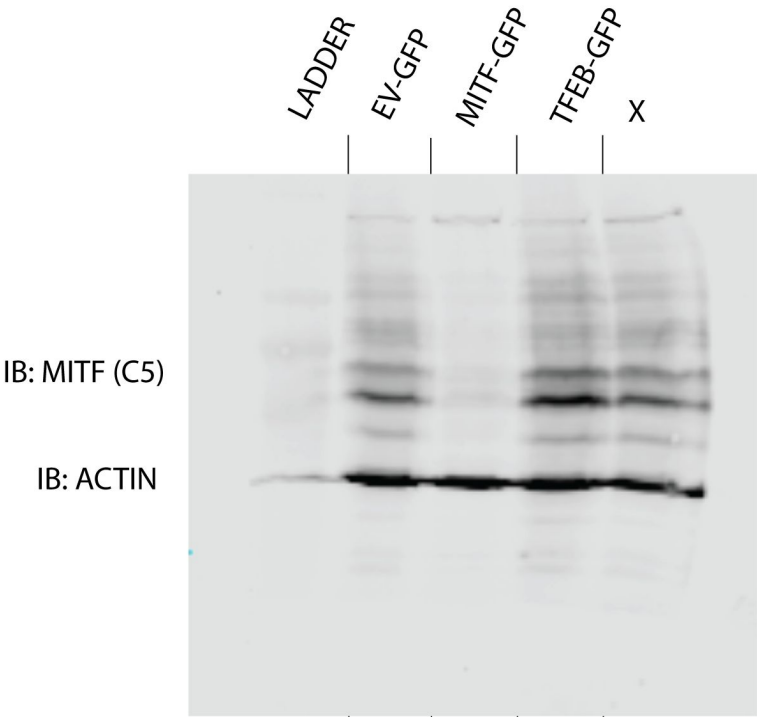

IB: TFEB

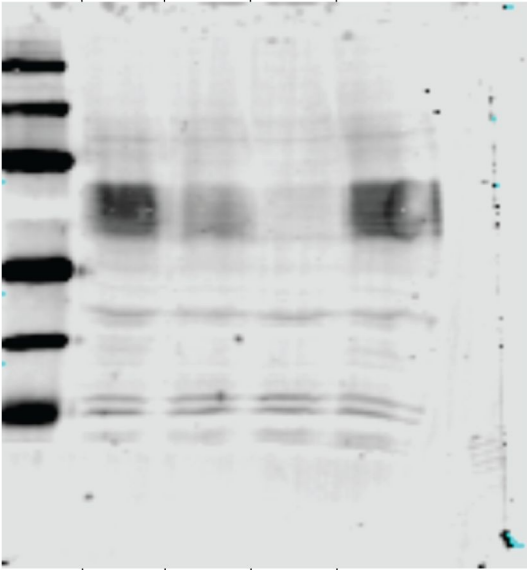

Figure 4C.

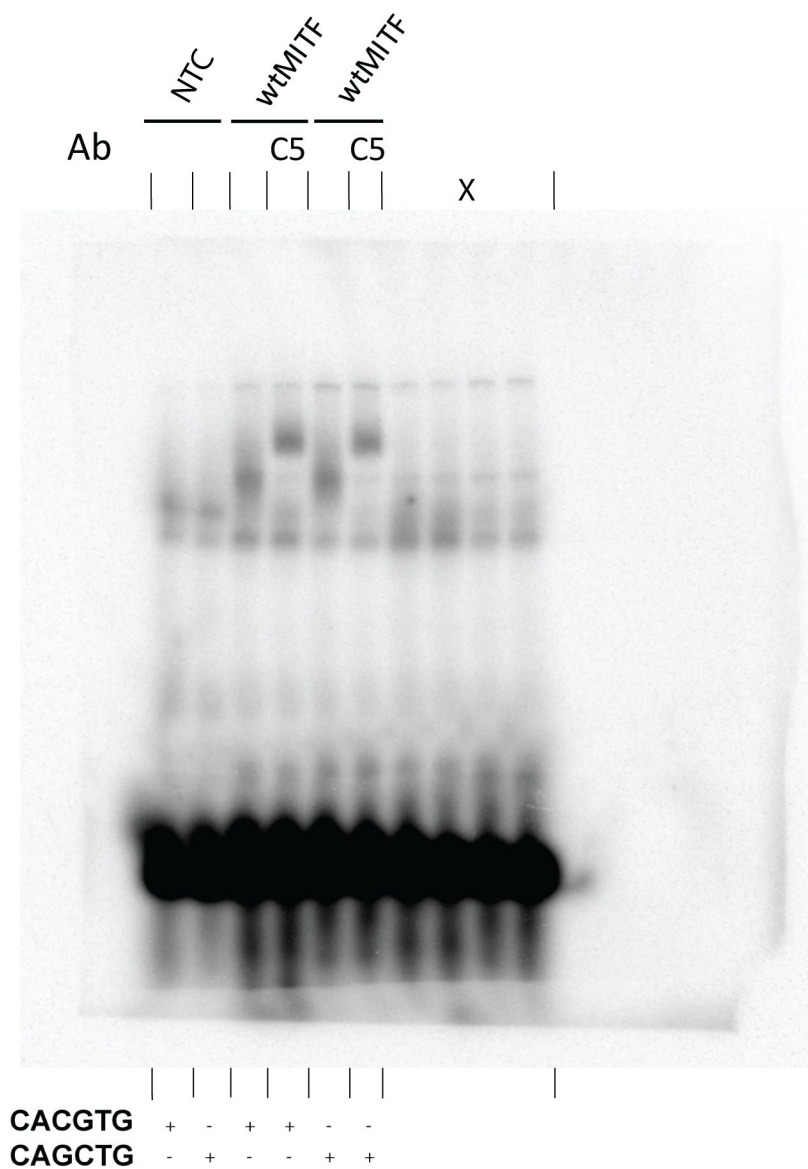

Supplement: S1 File — (PDF) [file pone.0238546.s009.pdf]
