# Supplementary material for: MITF and TFEB cross-regulation in melanoma cells
Source: PLoS One. 2020 Sep 3;15(9):e0238546. doi: 10.1371/journal.pone.0238546 (PMC7470386; doi:10.1371/journal.pone.0238546)
Supplement: Attachment — Submitted filename: Response to Reviewers.doc [file pone.0238546.s010.doc]

July 6, 2020

Dear editor,

We thank PLOS One for the opportunity to resubmit our manuscript after addressing the concerns of reviewers and editor. We also thank the reviewers for their critical comments and analysis. Following are the specifics of the changes we made:

1. We have made sure that the manuscript fits the PLOS ONE style requirements and renamed the files appropriately.
2. The uncropped and unadjusted images are provided for all images as supporting information.
3. The data previously referred to as “data not shown” is now available on Figshare (DOI: [10.6084/m9.figshare.12568646](https://doi.org/10.6084/m9.figshare.12568646)).

Reviewer #2:

***Comment 1****: In the final sentence of the manuscript “… improving our understanding of their role in healthy tissue …” does not consider what the expression status of the MiT-TFE family may be in normal melanocytic cells. The authors may consider if there are any databases examining expression of these genes in different tissues could be consulted e.g. Melanocytes in the skin--comparative whole transcriptome analysis of main skin cell types. Reemann P, Reimann E, Ilmjärv S, Porosaar O, Silm H, Jaks V, Vasar E, Kingo K, Kõks S.PLoS One. 2014 Dec 29;9(12):e115717. This data was extracted from.*

*S1 Table. RPKM values of genes we detected in MC, KC, FB and the whole skin.*

*Melanocytes*

*genes MC_1 MC_2 MC_3 MC_4*

*ENSG00000187098 199.197 156.316 191.509 167.668 MITF*

*ENSG00000112561 0.400417 1.12329 0.798733 0.35943 TFEB*

*ENSG00000068323 1.13973 1.78558 1.92091 1.13326 TFE3*

*ENSG00000105967 0.0451891 0.048543 0.0621599 0.0607503 TFEC*

We have consulted our own array-based gene expression data where the expression of MITF, TFEB and TFE3 was determined in melanocytes and melanoma cells. We have introduced the following into the results section, page 6, lines 72-76:

“Furthermore, analysis of gene expression in 23 human melanoma cell lines as well as in normal human epidermal melanocytes (NHEM) using a microarray platform revealed that the expression of TFEB and TFE3 was roughly 50-fold lower than that of MITF, whereas expression of TFEC mRNA was about 850-fold lower than that of MITF [21,31].”

We also added the following in the Discussion section, page 17, lines 317-317:

“MITF, TFEB and TFE3 are expressed to some extent across melanoma tumors and cell lines, whereas TFEC is not [21,31]. Similarly, TFEB and TFE3 mRNA expression can be detected in normal melanocytes, albeit at lower levels than MITF [31,41].”

***Comment 2.*** *The recent paper describing acetylation of MITF, in which the senior author of this manuscript is a co-author, should at least be cited. Some discussion of how MITF has a pool of low affinity binding sites that may keep it in the nucleus and how may this differ or be similar for the other MiT-TFE family, and effects of acetylation on other family members such as TFEB could be included.*

*Tuning Transcription Factor Availability through Acetylation-Mediated Genomic Redistribution.*

*Louphrasitthiphol P, Siddaway R, Loffreda A, Pogenberg V, Friedrichsen H, Schepsky A, Zeng Z, Lu M, Strub T, Freter R, Lisle R, Suer E, Thomas B, Schuster-Böckler B, Filippakopoulos P, Middleton M, Lu X, Patton EE, Davidson I, Lambert JP, Wilmanns M, Steingrímsson E, Mazza D, Goding CR.Mol Cell. 2020 Jun 4:S1097-2765(20)30345-2.*

We have entered a paragraph discussing this interesting study in the page 20, between lines 374-383, of the Discussion that reads the following:

“Recent studies have shown that the acetylation status of MITF impacts genomic occupancy as a means to modulate its transcriptional activity. Non-acetylated high DNA-binding-affinity MITF is able to bind a large pool of DNA loci including non-canonical degenerate motifs. In contrast, K243-acetylated MITF or the acetyl-mimetic K243Q mutant has low DNA-binding-affinity, yet robustly activates expression of melanocyte and melanoma target genes (54). It is possible that acetylation of MITF affects binding to the non-canonical CAGCTG motifs found in TFEB. Furthermore, mTORC1 has been shown to phosphorylate and positively regulate the p300 acetyltransferase (55), which in turn acetylates MITF (49, 54, 56), suggesting that the mTOR pathway might be capable of modulating not only the subcellular localization of the MiT-TFE factors, but also shift their genomic occupancy towards high-affinity sites.”

In addition, we reason that this study may be relevant to the observation that although the expression of TFEB is low in melanocytes and in some melanoma tumor and cell lines, the protein levels of this factor may be sufficient for binding and activation for a subet of high-affinity binding sites. This statement can be found in the Discussion section, pages 17-18 between lines 317-320:

“Although the expression of TFEB is low at the mRNA level, there may be sufficient protein in the cells to have major effects, especially taking into consideration the biological role of TFEB and recent findings regarding low affinity vs high affinity binding sites across the genome for a given transcription factor [42].“

Best regards

Eiríkur Steingrímsson, PhD
